# Clinical efficacy of various resuscitation fluids in the management of sepsis in postoperative surgical and trauma patients: a systematic review and meta­‐analysis

**DOI:** 10.20452/wiitm.2024.17900

**Published:** 2024-09-20

**Authors:** Yongjie Wang, Kewu Chen, Xiaolu Li, Jianing Guan

**Affiliations:** Department of Critical Care Medicine, Jilin Provincial People’s Hospital, Changchun, Jilin, China; Department of Emergency, Dazu Hospital of Chongqing Medical University, Chongqing, China; Intensive Care Unit, Chifeng Municipal Hospital of Inner Mongolia, Chifeng, China; Department of Emergency, Taizhou Central Hospital, Affiliated Hospital of Taizhou University, Taizhou Zhejiang, China

**Keywords:** critical hypotension, resuscitation fluids, sepsis, surgery, traumatic injury

## Abstract

**INTRODUCTION:**

Fluid resuscitation is the primary sepsis management strategy aimed at reducing mortality and achieving better treatment outcomes in critically hypotensive patients. Still, there are significant ambiguities regarding the most suitable fluid type that would ensure optimization of patient outcomes.

**AIM:**

The aim of this systematic review and meta-analysis was to assess the clinical effectiveness of different resuscitation fluids for sepsis management in critically hypotensive patients.

**MATERIALS AND METHODS:**

A systematic search of 4 electronic databases (PubMed, EMBASE, Scopus, and Cochrane Library) was conducted to identify relevant papers published in peer-reviewed journals since database inception until June 30, 2024. Odds ratios (ORs) with 95% CIs were calculated to evaluate the impact of individual resuscitation fluids on improvements in hemodynamic parameters and all-cause mortality. Heterogeneity was assessed using the Cochran Q, I^2^ statistic, and the appropriate *P* value.

**RESULTS:**

Our meta-analysis included 18 randomized controlled trials comparing the efficacy of different resuscitation fluids for sepsis management in 14 469 critically hypotensive patients. We found that Ringer’s lactate solution was more effective than saline in reducing mortality (OR, 0.53; 95% CI, 0.41–0.7; χ^2^= 3.47; degree of freedom [df] = 6; Z = 4.6; I^2^ = 0%; *P* <⁠0.001) and improving hemodynamic parameters (OR, 2.64; 95% CI, 2.45–2.86; χ^2^ = 48.36; df = 6; Z = 24.84; I^2^ = 18%; *P* <⁠0.001). However, saline was superior to albumin and hydroxyethyl starch in reaching these end points.

**CONCLUSION:**

We showed that in critically hypotensive septic patients, Ringer’s lactate solution reduces all-cause mortality and improves hemodynamic parameters more effectively than saline, hydroxyethyl starch, and albumin solutions.

## INTRODUCTION

Fluid resuscitation is the primary management strategy for critically hypotensive patients, and it has played a significant role in reducing mortality over the years.^1^^; ^^2^ Research indicates that early fluid resuscitation effectively prevents and restricts sepsis, inhibits prevalent multiorgan failure, improves microcirculation, and reduces the systemic inflammatory response.^3^^; ^^4^ Fluids are a fundamental component of resuscitation of critically hypotensive patients; however, fluid management strategies are subject to significant practice variations.^5^^; ^^6^ Clinical research has demonstrated that colloids and crystalloids have a distinct impact on a variety of critical physiological parameters. Fluid categories for the purpose of fluid resuscitation include blood products, colloids (eg, albumin, gelatin solutions, and hydroxyethyl starch [HES]), and crystalloids, including normal saline (NS) and Ringer’s lactate.^7^^; ^^8^^; ^^9^ Studies examining clinical outcomes of fluid resuscitation have reported reduced length of hospital stay, decreased mortality, improved tissue perfusion, and a reduced rate of systemic inflammatory response syndrome.^10^^;^ ^11^^;^ ^12^ Although fluid resuscitation has demonstrated exceptional treatment outcomes, there are still significant ambiguities regarding the most suitable fluid type and volumetric rates that would optimize patient outcomes. As the most frequently employed crystalloid, NS (0.9% sodium chloride, pH 7, and chloride content of 154 mmol/l) is believed to trigger hyperchloremic metabolic acidosis, which may have a direct impact on organ function and even survival.^13^ Similarly, colloid solutions are considered more effective than crystalloids in achieving an equivalent hemodynamic effect; however, they are also believed to induce changes in the immune response to critical illness.^14^ Furthermore, even though colloids (such as HES, gelatin, and albumin) have a prolonged half-life in plasma, they have been reported to impair glomerular filtration, and are associated with a higher risk of acute renal failure, anaphylactic shock, and death.^15^ Ringer’s lactate, a generally balanced crystalloid, has been recommended for aggressive fluid replacement therapy in critically hypotensive patients.^16^^; ^^17^

To address the evidence gap regarding the most suitable fluid type and volumetric rates that would optimize patient outcomes, the present study systematically compiled and evaluated evidence from 18 randomized controlled trials (RCTs)**;**^18^^; ^^19^^; ^^20^^; ^^21^^; ^^22^^; ^^23^^; ^^24^^; ^^25^^; ^^26^^; ^^27^^; ^^28^^; ^^29^^; ^^30^^; ^^31^^; ^^32^^; ^^33^^; ^^34^^; ^^35^ to determine the efficacy of various fluid types used for resuscitation purposes. We focused on studies analyzing critically hypotensive patients with sepsis and examining various fluid types (including colloids and crystalloids) to identify the most effective infusion for sepsis management. This systematic review and meta-analysis provides a comprehensive evaluation of existing evidence, making it a valuable contribution to the field. The findings may contribute to development of clinical practice guidelines and help improve patient outcomes in sepsis management, thus filling a significant knowledge gap in the field of fluid resuscitation.

## AIM 

The aim of this systematic review and meta-analysis was to assess the clinical effectiveness of different resuscitation fluids for sepsis management in critically hypotensive patients.

## MATERIALS AND METHODS 

### Search strategy and selection criteria 

This meta-analysis and systematic review complies with the Assessing the Methodological Quality of Systematic Reviews (AMSTAR^36^) and Preferred Reporting Items for Systematic Reviews and Meta-Analyses (PRISMA) guidelines.^37^ The efficacy of various resuscitation fluids in septic, surgical, and trauma patients was compared through a systematic review of relevant RCTs that were selected in accordance with predefined inclusion and exclusion criteria. A comprehensive search of the scientific literature databases (EMBASE, PubMed, Scopus, and Cochrane Library) for articles published since database inception until June 30, 2024 was conducted to identify the pertinent RCTs. The search terms used were: *resuscitation fluid* OR *fluid therapy* OR *volume replacement* OR *sepsis* OR *critically hypotensive patients* OR *septic disease* OR *injury* OR *surgical patients* OR *trauma patients* OR *hydroxyethyl starch* OR *HES* OR *gelatin* OR *saline* OR *albumin* OR *Ringer’s lactate* OR *crystalloids* OR *all-cause mortality* OR *organ dysfunction* OR *organ replacement therapy* OR *mortality* OR *bleeding* OR *death* OR *transfusion* OR *colloid* *substance* OR *hemodynamic parameters* OR *hemodialysis* OR *hemofiltration* OR *randomized controlled trial* OR *RCT* OR *systematic review* OR *meta-analysis*. Key words for agreement in both Medline and EMBASE databases were identified and evaluated using the Patient, Intervention, Comparison, Outcome (PICO) criteria.^38^ The specified key words were inserted into the title / abstract / key word field during the Scopus search. In the Cochrane database, the search terms *sepsis*, *critically hypotensive patients*, and *resuscitation fluid* were used. The PICO framework was employed to establish precise selection criteria. The letter “P” indicated patients who suffered from trauma or sepsis, or underwent surgery. The letter “I” represented the intervention group, while “C”, the control group. The improvement in hemodynamic parameters and all-cause mortality were the primary clinical outcomes, denoted by the letter “O”. Our meta-analysis only included RCTs. Additional potentially relevant papers were identified by conducting backward and forward citation monitoring of previously published meta-analyses and included studies. TABLE 1 illustrates the comprehensive database search strategy. The titles, abstracts, and full texts of potentially eligible articles were independently assessed by 2 reviewers (YJW and KWC). If required, a senior researcher was consulted, and any discrepancies between the 2 reviewers were resolved through discussion.

**TABLE 1:** Database search strategy

Database	Search strategy
Scopus	1 *Resuscitation fluid* OR *fluid therapy* OR *volume replacement* OR *sepsis* OR *critically hypotensive patients* OR *septic disease* OR *injury* OR *surgical patients* OR *trauma patients* OR *hydroxyethyl starch* OR *HES* OR *gelatin* OR *saline* OR *albumin* OR *Ringer’s lactate* OR *crystalloids*
	2 *All-cause mortality* OR *organ dysfunction* OR *organ replacement therapy* OR *mortality* OR *bleeding* OR *death* OR *transfusion* OR *colloid substance* OR *hemodynamic parameters* OR *hemodialysis* OR *hemofiltration* OR *randomized controlled trial* OR *RCT* OR *systematic review* OR *meta-analysis*
	3 #1 and #2
PubMed	1 R*esuscitation fluid* OR *fluid therapy* (MeSH terms**)** OR *volume replacement* (all fields) OR *sepsis* (MeSH terms) OR *critically hypotensive patients* (all fields) OR *septic disease* (all fields) OR *injury* (all fields) OR *surgical patients* (all fields) OR *trauma patients* (all fields) OR *hydroxyethyl starch* (all fields) OR *HES* (all fields) OR *gelatin* (all fields) OR *saline* (all fields) OR *albumin* (all fields) OR *Ringer’s lactate* (all fields) OR *crystalloids* (all fields)
	2 A*ll-cause mortality* (MeSH terms) OR *organ dysfunction* (all fields) OR *organ replacement therapy* (all fields) OR *mortality* (all fields) OR *bleeding* (all fields) OR *death* (all fields) OR *transfusion* (all fields) OR *colloid substance* (all fields) OR *hemodynamic parameters* (all fields) OR *hemodialysis* (all fields) OR *hemofiltration* (all fields) OR *randomized controlled trial* (all fields) OR *RCT* (all fields) OR *systematic review* (all fields) OR *meta-analysis* (all fields)
	3 #1 and #2
EMBASE	1 R*esuscitation fluid* / exp OR *fluid therapy* / exp OR *volume replacement* / exp OR *sepsis* / exp OR *critically hypotensive patients* / exp OR *septic disease* / exp OR *injury* / exp OR *surgical patients* / exp OR *trauma patients* / exp OR *hydroxyethyl starch* / exp OR *HES */ exp OR *gelatin* / exp OR *saline* / exp OR *albumin* / exp OR *Ringer’s lactate* / exp OR *crystalloid*
	2 *All-cause mortality* / exp OR *organ dysfunction* / exp OR *organ replacement therapy* / exp OR *mortality */ exp OR *bleeding* / exp OR *death* / exp OR *transfusion* / exp OR *colloid substance* / exp OR *hemodynamic parameters* / exp OR *hemodialysis* / exp OR *hemofiltration* / exp OR *randomized controlled trial* / exp OR *RCT* / exp OR *systematic review* / exp OR *meta-analysis* / exp
	3 #1 and #2
Cochrane library	1 (*Resuscitation fluid*): ti, ab, kw OR (*fluid therapy*): ti, ab, kw OR (*volume replacement*): ti, ab, kw OR (*sepsis*): ti, ab, kw OR (*critically hypotensive patients*): ti, ab, kw OR (*septic disease*): ti, ab, kw OR (*injury*): ti, ab, kw OR (*surgical patients*): ti, ab, kw OR (*trauma patients*): ti, ab, kw OR (*hydroxyethyl starch*): ti, ab, kw OR (*HES*): ti, ab, kw OR (*gelatin*): ti, ab, kw OR (*saline*): ti, ab, kw OR (*albumin*): ti, ab, kw OR (*Ringer’s lactate*): ti, ab, kw OR (*crystalloid*): ti, ab, kw (word variations have been searched)
	2 (*All-cause mortality*): ti, ab, kw OR (*organ dysfunction*): ti, ab, kw OR (*organ replacement therapy*): ti, ab, kw OR (*mortality*): ti, ab, kw OR (*bleeding*): ti, ab, kw OR (*death*): ti, ab, kw OR (*transfusion*): ti, ab, kw OR (*colloid substance*): ti, ab, kw OR (*hemodynamic parameters*): ti, ab, kw OR (*hemodialysis*): ti, ab, kw OR (*hemofiltration*): ti, ab, kw OR (*randomized controlled trials*): ti, ab, kw OR (*RCT*): ti, ab, kw OR (*systematic review*): ti, ab, kw OR (*meta-analysis*): ti, ab, kw (word variations have been searched)
	3 #1 and #2

Abbreviations: exp, explosion in Emtree—searching of selected subject terms and related subjects; MeSH, medical subject headings; ti, ab, kw, title or abstract or keyword field

### Study selection and data extraction

The study included RCTs that provided comparative data on clinical effectiveness of different resuscitation fluids in critically hypotensive patients. There were no limitations with respect to the year or language of publication. The specific inclusion criteria were as follows: 1) RCT design; 2) analysis of critically hypotensive patients with sepsis; 3) participants aged at least 18 years; 4) focus on improved patient hemodynamic parameters and all-cause mortality; and 5) full-text papers with sufficient data for generating a 2 × 2 table.

Non-RCTs (case series, case-control studies, and cohort studies), narrative or expert evaluations, animal studies or trials, trials involving children, and studies that did not analyze critically hypotensive patients were excluded from analysis, as were outdated, anecdotal, or entirely expert-based bibliographic references. Patient demographic profiles and event data from the included studies were independently collected by 2 researchers using a predetermined form. The extracted information included author names and year of publication, country of study, number of included centers, total number of patients, age of participants, number of septic patients, types of resuscitation fluids used in the control and intervention arms, inclusion-exclusion criteria of the RCTs, and primary outcomes. The authors were contacted to provide supplementary information when their data were deemed insufficient or ambiguous.

### Risk of bias assessment

We employed a standardized questionnaire to evaluate the included studies for possible bias. Separate risk of bias assessments were carried out by 2 researchers (YJW and KWC) using the Cochrane risk-of-bias tool, version 2.^39^ The tool comprises 5 components: randomization-induced bias, bias resulting from variations from intended interventions, bias due to missing outcome information, bias during outcome evaluation, and bias in selecting reported results. A third reviewer (XL) assumed the role of an arbiter to settle any arising disputes. Any potential bias was evaluated and classified as either “uncertain risk,” “high risk,” or “low risk.” Small-study effects and publication bias were evaluated using a comparison-adjusted funnel plot.^40^ The significance of bias was evaluated with the Egger regression test^41^ using MedCalc software.^42^

### Statistical analysis

Statistical analysis was performed using Review Manager (RevMan) software, version 5.4.^43^ For each study, odds ratios (ORs) and 95% CIs^44^ were computed to assess binary outcomes. The DerSimonian–Laird method^45^ was employed to calculate the ORs using a 2 × 2 Table^46^ illustrating event data. During quantitative evaluation, we excluded studies that did not report the use of any resuscitation fluid or the presence of septic patients in either group. We designed forest plots^47^ to evaluate the influence of different outcome determinants using the Mantel–Haenszel fixed-effects model, since there was no interstudy heterogeneity. Statistical parameters, such as the I^2^ value,^48^ χ^2^ value,^49^ Z value,^50^ and *P* value^51^ were used to assess heterogeneity. The *P* value below 0.05 was deemed significant. We conducted a subgroup analysis to evaluate the efficacy of various resuscitation fluids, including Ringer’s lactate vs saline, Ringer’s lactate vs HES, saline vs albumin, and saline vs HES, in terms of the change in primary outcomes, such as improvement in hemodynamic parameters and all-cause mortality. A hierarchical summary receiver-operating characteristic curve (HSROC) plot^52^ was designed to check the test accuracy of the included RCTs.

### Ethics

An ethics statement is not applicable as this study is based exclusively on published literature.

## RESULTS 

### Study selection outcomes 

**FIGURE 1:**
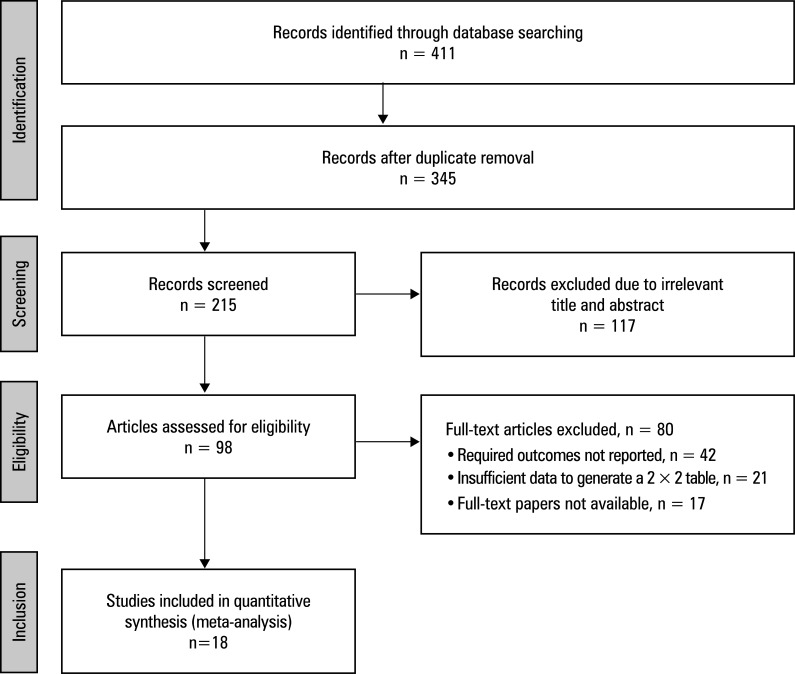
Preferred Reporting Items for Systematic Reviews and Meta-Analysis (PRISMA) study flow diagram

This study was conducted through an exhaustive electronic search of 4 databases. Consequently, 411 studies were identified as meeting the inclusion criteria specified in the PICO framework. A total of 345 articles were selected for review, while 66 were excluded due to duplicate content. A subsequent assessment of eligibility was conducted for 215 papers following additional screening. Of those, 117 were excluded due to irrelevant titles and abstracts, and the remaining 98 were subjected to a further assessment. However, after applying the inclusion–exclusion criteria, 80 studies were found ineligible and were excluded. This was primarily due to a lack of required outcomes, insufficient data to generate 2 × 2 tables, or unavailability of full-text papers. Finally, 18 RCTs that met the predetermined inclusion–exclusion criteria were included in this meta-analysis, as illustrated in the study flow diagram based on the PRISMA guidelines (FIGURE 1). Overall, they presented data of 14 469 patients aged 18 years or older. The efficacy of Ringer’s lactate solution vs saline was compared in 7 of the 18 included studies.^19^^; ^^23^^; ^^24^^; ^^28^^; ^^30^^; ^^32^^; ^^35^ Additionally, 4 studies compared saline with albumin,^21^^; ^^22^^; ^^26^^; ^^27^ 4 compared saline with HES, ^25^^; ^^29^^; ^^31^^; ^^34^ and the remaining 3 studies compared Ringer’s lactate with HES. ^18^^; ^^20^^; ^^33^ A comprehensive overview of patient demographic characteristics is provided in TABLE 2. All the included studies provided event data for the generation of 2 × 2 tables to facilitate this meta-analysis.

**TABLE 2:** Characteristics of the included randomized controlled trials (continued on the next page)

Author	Country of study	Included centers, n	Patients, n	Patient age, y	Sepsis patients, n	Control arm	Intervention arm	Inclusion criteria	Exclusion criteria	parameters and 90­‐d mortality Primary outcomes
Angsubhakorn et al^18^	Thailand	1	34	46	34	HES	RL	Sepsis, multiple traumas, or other causes of hypovolemic shock	Age <18 y, pregnancy, admission to the ICU following transplantation surgery, previous fluid resuscitation during the current admission	Hemodynamic parameters and 90­‐day mortality
Annane et al (CRISTAL)^19^	Worldwide	57	2857	63	1553	Saline	RL	Sepsis, multiple traumas, or other causes of hypovolemic shock	Chronic organ failure requiring replacement therapies, or patients with diabetes mellitus or known aortic aneurysm	Hemodynamic parameters and 90­‐day mortality
Brunkhorst et al (VISEP)^20^	Germany	18	537	65	537	HES	RL	Severe sepsis or septic shock	Patients with hemodynamic instability requiring aggressive resuscitation during the first 100 minutes	Hemodynamic parameters and 90­‐day mortality
Caironi et al (ALBIOS)^21^	Italy	100	1818	69	1818	Saline	Albumin	Severe sepsis or septic shock	Age <18 y, pregnancy, admission to the ICU following transplantation surgery, previous fluid resuscitation during the current admission	Hemodynamic parameters and 90­‐day mortality
Charpentier et al (EARSS)^22^	France	29	798	66	798	Saline	Albumin	Suspected hypovolemia, septic shock and severe respiratory failure	Chronic organ failure requiring replacement therapies, or patients with diabetes mellitus or known aortic aneurysm	Hemodynamic parameters and 28­‐day mortality
Cuellar et al^23^	Mexico	1	88	50	88	Saline	RL	Suspected hypovolemia, septic shock, and severe respiratory failure	Chronic organ failure requiring replacement therapies, or patients with diabetes mellitus or known aortic aneurysm	Hemodynamic parameters and 30­‐day mortality
De­‐Madaria et al^24^	Spain	18	744	60	249	Saline	RL	Suspected hypovolemia, septic shock, and severe respiratory failure	Chronic organ failure requiring replacement therapies, or patients with diabetes mellitus or known aortic aneurysm	Hemodynamic parameters and 90­‐day mortality
Dubin et al^25^	Argentina	2	20	65	20	HES	Saline	Severe sepsis or septic shock	Chronic organ failure requiring replacement therapies, or patients with diabetes mellitus or known aortic aneurysm	Hemodynamic parameters and 31­‐day mortality
Finfer et al (SAFE)^26^	Australia and New Zealand	16	1218	58	1218	Saline	Albumin	Septic shock and severe respiratory failure	Age <18 y, pregnancy, admission to the ICU following transplantation surgery, previous fluid resuscitation during the current admission	Hemodynamic parameters and 28­‐day mortality
Friedman et al^27^	Belgium	1	34	66	34	Saline	Albumin	Suspected hypovolemia, septic shock, and severe respiratory failure	Hemodynamic instability requiring aggressive resuscitation during the first 100 minutes	Hemodynamic parameters and in­ hospital mortality
Li et al^28^	China	11	912	50	912	Saline	RL	Suspected hypovolemia, septic shock, and severe respiratory failure	Age <18 y, admission to the ICU following cardiac surgery, body burn, liver transplantation surgery, previous fluid resuscitation during the current admission	Hemodynamic parameters and 90­‐day mortality
Li et al^29^	China		60	45	60	HES	Saline	Septic shock and severe respiratory failure	Age <18 y, pregnancy, admission to the ICU following transplantation surgery, previous fluid resuscitation during the current admission	Hemodynamic parameters and 28­‐day mortality
Semler et al (SMART)^30^	United States	1	2336	58	2336	Saline	RL	Severe sepsis or septic shock	Chronic organ failure requiring replacement therapies, or patients with diabetes mellitus or known aortic aneurysm	Hemodynamic parameters and 30­‐day mortality
McIntyre et al (FINESS)^31^	Canada and New Zealand	4	40	64	40	HES	Saline	Sepsis, multiple traumas, or other causes of hypovolemic shock	Age <18 y, other forms of shock, chronic renal failure requiring dialysis, pregnancy, previous admission to the ICU with septic shock during the current hospitalization	Hemodynamic parameters and 28­‐day mortality
Messallam et al^32^	United States	1	310	47	208	Saline	RL	Sepsis, multiple traumas, or other cause of hypovolemic shock	Age <18 y, pregnacyt, admission to the ICU following transplantation surgery, previous fluid resuscitation during the current admission	Hemodynamic parameters and 30­‐days mortality
Molnar et al^33^	United Kingdom	1	30	56	30	HES	RL	Septic shock and severe respiratory failure	Chronic organ failure requiring replacement therapies, or patients with diabetes mellitus or with known aortic aneurysm	Hemodynamic parameters and in­ hospital mortality
Myburgh et al (CHEST)^34^	Australia and New Zealand	32	1937	63	1937	HES	Saline	Sepsis, multiple traumas, or other causes of hypovolemic shock	Age <18 y, pregnancy, admission to the ICU following transplantation surgery, previous fluid resuscitation during the current admission	Hemodynamic parameters and 90­‐day mortality
Perner et al (6S)^35^	Scandinavia	26	804	67	798	Saline	RL	Severe sepsis or septic shock	Age <18 y, pregnancy, admission to the ICU following transplantation surgery, previous fluid resuscitation during the current admission	Hemodynamic parameters and 90­‐day mortality

Abbreviations: HES, hydroxyethyl starch; ICU, intensive care unit; RL, Ringer’s lactate

### Risk of bias assessment

**FIGURE 2:**
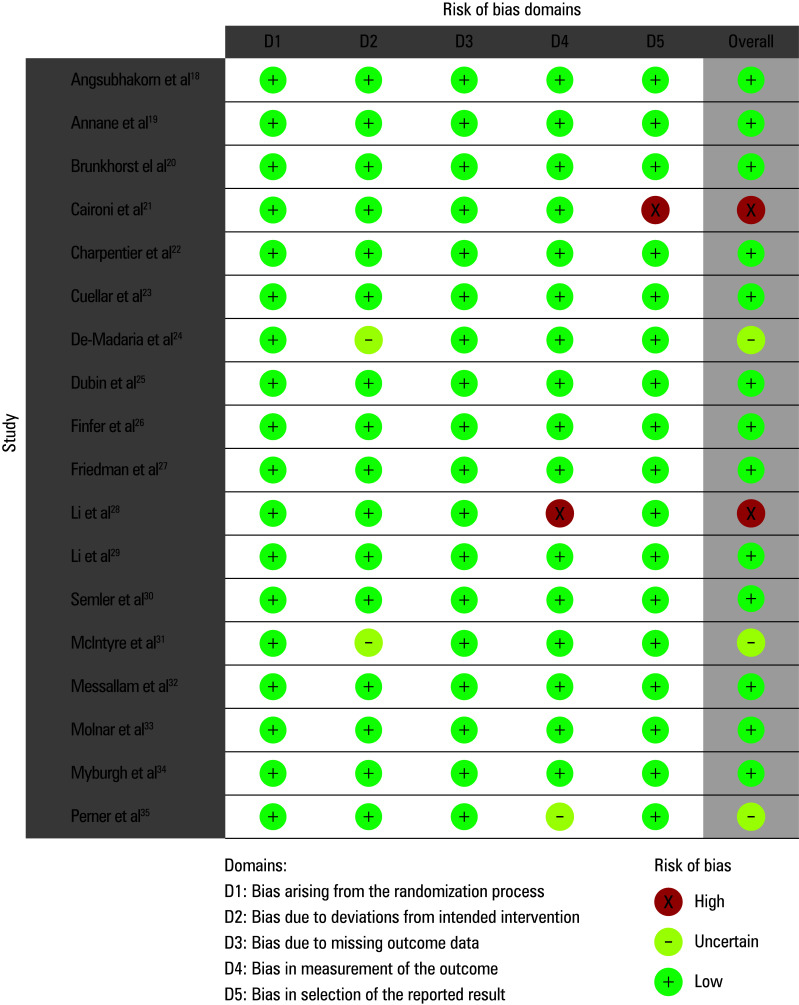
Traffic light plot for risk of bias assessment

**FIGURE 3:**
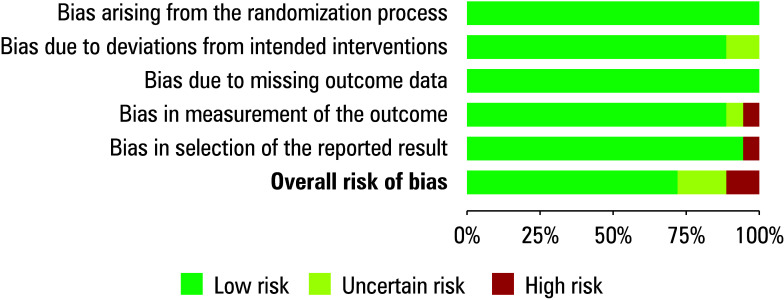
Risk of bias summary plot

We conducted a risk of bias evaluation to determine the study’s overall quality score. TABLE 3 displays the results of the risk of bias assessment for each of the 18 included RCTs. The meta-analysis showed a low risk of bias, as indicated by the traffic light plot for bias assessment shown in FIGURE 2 and the summary plot shown in FIGURE 3. Out of the 18 RCTs, 13 had a low risk of bias. The risk of bias for 3 RCTs was moderate due to issues related to deviations from the intended intervention or bias in the measurement of the outcome. The remaining 2 RCTs showed a high risk of bias, specifically in the measurement of the outcome and the selection of reported results, respectively

**TABLE 3:** Risk assessment of the included studies using the Cochrane risk­‐of­‐bias tool

Signaling question	Angsubhakorn et al^18^	Annane et al^19^	Brunkhorst et al^20^	Caironi et al^21^	Charpentier et al^22^	Cuellar et al^23^	De­-Madaria et al^24^	Dubin et al^25^	Finfer et al^26^	Friedman et al^27^	Li et al^28^	Li et al^29^	Semler et al^30^	McIntyre et al^31^	Messallam et al^32^	Molnar et al^33^	Myburgh et al^34^	Perner et al^35^
Was a consecutive or random sample of patients enrolled?	Y	Y	Y	Y	Y	Y	Y	Y	Y	Y	Y	Y	Y	Y	Y	Y	Y	Y
Did the study avoid inappropriate exclusions?	Y	Y	Y	Y	Y	Y	Y	Y	Y	Y	Y	Y	Y	Y	Y	Y	Y	Y
Did all patients receive the same reference standard?	Y	Y	Y	Y	Y	Y	Y	Y	Y	Y	Y	Y	Y	Y	Y	Y	Y	Y
Were all patients included in the analysis?	N	N	N	N	N	N	N	N	N	N	N	N	N	N	N	N	N	N
Was the sample frame appropriate to address the target population?	Y	Y	Y	Y	Y	Y	Y	Y	Y	Y	Y	Y	Y	Y	Y	Y	Y	Y
Were the study participants sampled in an appropriate way?	Y	Y	Y	Y	Y	Y	Y	Y	Y	Y	Y	Y	Y	Y	Y	Y	Y	Y
Were the study participants and the setting described in detail?	Y	Y	Y	Y	Y	Y	Y	Y	Y	Y	Y	Y	Y	Y	Y	Y	Y	Y
Were valid methods used for identification of the condition?	Y	Y	Y	Y	Y	Y	Y	Y	Y	Y	Y	Y	Y	Y	Y	Y	Y	Y
Was the condition measured in a standard, reliable way for all participants?	Y	Y	Y	Y	Y	Y	Y	Y	Y	Y	Y	Y	Y	Y	Y	Y	Y	Y

Abbreviations: Y, yes; N, no

### Subgroup analysis assessing the effect of different resuscitation fluids on all-cause mortality

**FIGURE 4:**
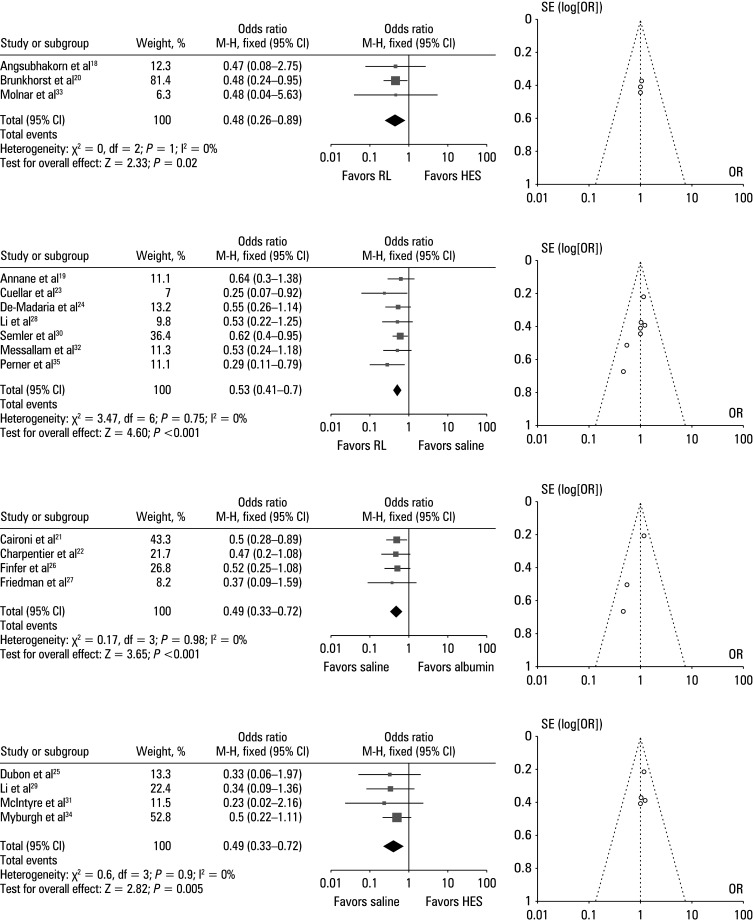
Subgroup analysis illustrating forest plots and funnel plots for all­‐cause mortality; A – Ringer’s lactate (RL) vs hydroxyethyl starch (HES); B – RL vs saline; C – saline vs albumin; D – saline vs HES

Abbreviations: df, degrees of freedom; M-H, Mantel–Haenszel fixed-effects model

To determine the effect of different resuscitation fluids on all-cause mortality, we used the event data extracted from the included trials to calculate the ORs. FIGURE 4A presents a comparison of the effect of Ringer’s lactate solution and HES, demonstrating that the odds of all-cause mortality were lower for the group treated with the Ringer’s lactate solution (OR, 0.48; 95% CI, 0.26–0.89; χ^2^ = 0; df = 2; Z = 2.33; I^2^ = 0%; *P* = 0.02). Moreover, the symmetrical funnel plot and insignificant results of the Egger test (*P* = 0.23) indicated a low likelihood of publication bias. A comparison between Ringer’s lactate solution and saline illustrated in FIGURE 4B shows that the likelihood of mortality was lower for Ringer’s lactate, with the OR value of 0.53 (95% CI, 0.41–0.7; χ^2^ = 3.47; df = 6; Z = 4.6; I^2^ = 0%; *P *<⁠0.001). Additionally, the symmetrical shape of the funnel plot and *P* value of 0.26 indicated a low risk of publication bias. The compared effects of saline solution and albumin depicted in FIGURE 4C indicate a lower rate of all-cause mortality in the saline-treated group than in the albumin-treated patients, with the OR value of 0.49 (95% CI, 0.33–0.72; χ^2^ = 0.17; df = 3; Z = 3.65; I^2^ = 0%; *P* <⁠0.001), and a low risk of publication bias, with a symmetrical funnel plot and *P* = 0.3 for the Egger test. A comparison between the saline solution and HES presented in FIGURE 4D demonstrated that the likelihood of mortality was lower in the saline-treated group, with the OR value of 0.41 (95% CI, 0.22–0.76; χ^2^ = 0.6; df = 3; Z = 2.82; I^2^ = 0%; *P* = 0.005) and low publication bias, as reflected by a symmetrical funnel plot and *P* = 0.34 for the Egger test.

### Subgroup analysis assessing the effect of different resuscitation fluids on hemodynamic parameters

**FIGURE 5:**
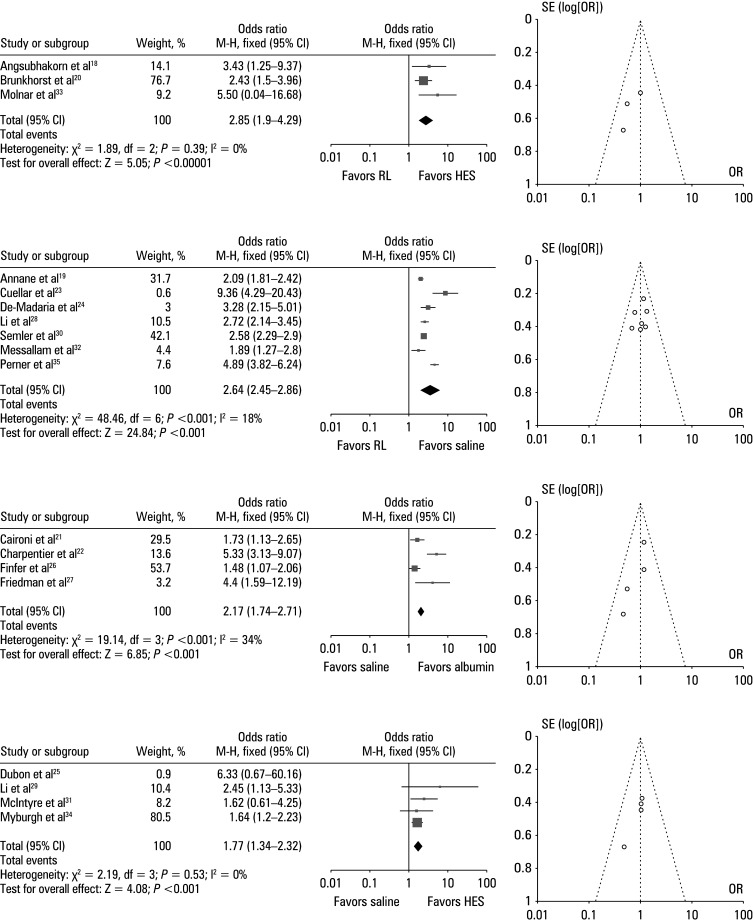
Subgroup analysis illustrating forest plots and funnel plots for improvement in hemodynamic parameters

To ascertain the impact of various resuscitation fluids on hemodynamic parameters, we computed the ORs in the intervention and control groups using the event data from the included RCTs. FIGURE 5A illustrates a comparison of the effect of Ringer’s lactate solution and HES. The odds of improvement in hemodynamic parameters were higher in the Ringer’s lactate solution–treated group (OR, 2.85; 95% CI, 1.9–4.29; χ^2^ = 1.89; df = 2; Z = 5.05; I^2^ = 0%; *P* <⁠0.001). In addition, a symmetrical funnel plot and insignificant *P* value for the Egger test (*P* = 0.18) suggested a low probability of publication bias. A comparison of Ringer’s lactate solution with saline is depicted in FIGURE 5B. The likelihood of improvement in hemodynamic parameters was higher for Ringer’s lactate than for saline, with the OR value of 2.64 (95% CI, 2.45–2.86; χ^2^ = 48.36; df = 6; Z = 24.84; I^2^ = 18%; *P* <⁠0.001). Furthermore, a symmetrical shape of the funnel plot and a *P* value of 0.33 suggested a low risk of publication bias. The comparative effects of saline solution and albumin are depicted in FIGURE 5C, which shows a more notable improvement in hemodynamic parameters among the saline-treated patients than in those treated with albumin (OR, 2.17; 95% CI, 1.74–2.71; χ^2^ = 19.14; df = 3; Z = 6.85, I^2^ = 34%; *P* <⁠0.001). The risk of publication bias was low, with a symmetrical funnel plot and *P* = 0.397 for the Egger test. FIGURE 5D illustrates a comparison of saline solution with HES, demonstrating that the likelihood of improvement in hemodynamic parameters was higher in the saline-treated group, with the OR value of 1.77 (95% CI, 1.34–2.32; χ^2 ^= 2.19; df = 3; Z = 4.08; I^2^ = 0%; *P *<⁠0.001) and low publication bias, as reflected by a symmetrical funnel plot and *P* = 0.22 for the Egger test.

**A** – Ringer’s lactate (RL) vs hydroxyethyl starch (HES); **B** – RL vs saline; **C** – saline vs albumin; **D** – saline vs HES

### Hierarchical summary receiver operating characteristic curve plot for test accuracy of included studies

**FIGURE 6:**
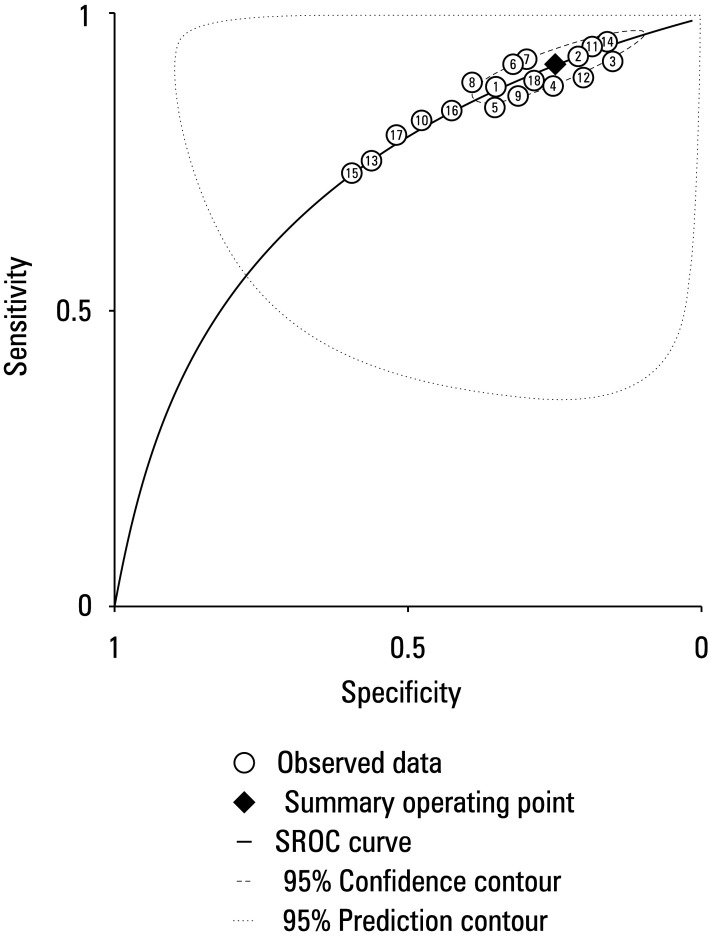
Hierarchical summary receiver operating characteristic (SROC) plot. Each examined study is represented by a circle, with the point estimate corresponding to cumulative sensitivity. Specificity is represented by a square, whereas the dashed line indicates the related 95% CI. The curve is represented as a linear line. The curve that summarizes the overall diagnostic accuracy is depicted by the regression line.

A HSROC diagram used to assess the test accuracy of each study is presented in FIGURE 6. The test accuracy of all the included studies was high, as indicated by the clustering of all data points in the upper left corner, whereas the sensitivity values were nearly 1 and the specificity values were almost 0. The area under the curve of the HSROC was 0.9 (95% CI, 0.7–0.95), which reflects inherent reliability of the diagnostic tests.

## DISCUSSION 

Sepsis is a severe condition in which the body’s immune defense mechanisms fail to adequately respond to an infection. The body attacks its own tissues, which often results in organ dysfunction.^53^^; ^^54^ Toxins produced by bacteria can impair the heart’s capacity to pump blood to organs, which is conducive to blood pressure lowering, if left untreated. Sepsis may lead to septic shock^55^ and critical hypotension that can potentially harm the liver, kidneys, lungs, and other organs.^56^ Fluid management in critically hypotensive septic patients has been the subject of close attention in recent years.^57^^; ^^58^ Fluid resuscitation involves administration of a variety of drug types and formulations, as well as selection of adequate infusion schedules and doses. Patient outcomes are directly affected by these parameters.^59^^; ^^60^ Consequently, a comprehensive understanding of the therapeutic benefits and adverse effects of these fluids is critical to determine the most suitable form of their administration. Resuscitation fluids play a crucial role in sepsis treatment, as they replenish intravascular volume, restore blood pressure, and maintain tissue perfusion. Crystalloids, such as NS and Ringer’s lactate, help increase intravascular volume and sodium content, raising blood pressure and improving cardiac output. Colloids, such as albumin and HES, induce oncotic pressure, whereby the fluid moves fluid into the intravascular space and blood volume is maintained. Additionally, some colloids may modulate the immune response and reduce inflammation. Blood products, including fresh frozen plasma and packed red blood cells, help restore blood’s oxygen-carrying capacity and coagulation factors. These fluids also contribute to mitigating the systemic inflammatory response syndrome and multiorgan dysfunction associated with sepsis. By replenishing fluids and electrolytes, as well as enhancing oxygen-carrying capacity of blood, resuscitation fluids help stabilize patient hemodynamic status, thereby reducing the risk of organ failure and mortality.^61^^; ^^62^^; ^^63^^; ^^64^ The choice of fluid depends on specific patient needs, and a balanced approach often involves a combination of crystalloids, colloids, and blood products to achieve optimal resuscitation.

It has been reported that NS is the most frequently used crystalloid worldwide for the management of sepsis in critically hypotensive patients. However, it is known to cause hyperchloremic acidosis that can impair renal function and increase the risk of infections.^65^^; ^^66^ We found that Ringer’s lactate solution was more effective than saline in reducing mortality (OR, 0.53; 95% CI, 0.41–0.7) and improving hemodynamic parameters (OR, 2.64; 95% CI, 2.45–2.86). However, saline was shown to outperform albumin and HES in this respect. Ringer’s lactate solution is a balanced or buffered solution used for fluid replacement. It is a type of isotonic, crystalloid fluid that is composed of sodium, chloride, potassium, calcium, and lactate in the form of sodium lactate. It has an osmolarity of 273 mOsm/l and a pH of approximately 6.5. In contrast, the osmolarity of NS is approximately 286 mOsm/l. Ringer’s lactate is primarily used in aggressive volume resuscitation of patients with blood loss or burn injuries, and in clinical scenarios such as acute pancreatitis and sepsis.^67^^; ^^68^^; ^^69^ Ringer’s lactate solution helps achieve volume resuscitation by increasing perfusion and expanding intravascular volume. It also provides the body with sodium lactate, a bioenergetic fuel that the human body is designed to metabolize under ischemic conditions, which reduces cellular mortality.^70^

Our findings concur with previous meta-analyses and sepsis guidelines^71^^; ^^72^ that recommend crystalloids as the preferred resuscitation fluids. In their systematic review and sequential network meta-analyses of resuscitation fluid types in sepsis, surgical, and trauma patients, Tseng et al^73^ concluded that balanced crystalloids and albumin reduced mortality more effectively than laevorotatory HES (L-HES) and saline in septic patients. However, in traumatic brain injury patients, saline or L-HES performed better than albumin or balanced crystalloids. In a similar vein, Liu et al^74^ reported that balanced crystalloids were probably the best option for a majority of critically ill patients who required fluid resuscitation. Nevertheless, utilization of HES was linked to an elevated risk of renal replacement therapy and an elevated incidence of acute kidney injury. These findings confirmed that for septic and surgical patients, balanced crystalloids, specifically Ringer’s lactate, were the most effective resuscitation fluids. They have been demonstrated to result in lower mortality rates, improved hemodynamic parameters, reduced risks of acute kidney injury, and lower blood transfusion volumes, as compared with saline and L-HES. However, Meyhoff et al^75^ found very little low-quality evidence supporting the decision on high vs low volumes of intravenous fluid therapy in adults with sepsis. Similarly, Meyhoff et al^76^ concluded that among adult patients with septic shock treated in an intensive care unit, intravenous fluid restriction did not result in fewer deaths at 90 days than standard intravenous fluid therapy. Furthermore, Zhang et al^77^ documented the possible advantages and effectiveness of the goal-directed fluid therapy approach, led by the Vigileo-FloTrac system, on the intestinal mucosal barrier for fluid infusion in elderly patients diagnosed with colorectal cancer.

### Limitations 

Certain limitations of our study need to be acknowledged. It is of vital importance to recognize the potential selection bias of our research, as a substantial number of studies were excluded. Secondly, our meta-analysis comprised only 18 studies of considerable heterogeneity related to varying fluid infusion quantities, fluid resuscitation procedures, and fluid intervention durations. Thirdly, the combined trials included distinct critically hypotensive septic patient populations that required immediate volume resuscitation. This factor had a potentially significant impact on the variation observed between the trials. Furthermore, variability in fluid administration protocols and variation of septic and critically hypotensive patients across the included studies also might have affected the results. Lastly, this study comprised a restricted number of RCTs and a small sample size for precise comparisons. Therefore, it is crucial to conduct further research on a larger sample and a larger number of RCTs in order to ascertain the optimal resuscitation fluid for sepsis management in critically hypotensive patients.

## CONCLUSIONS 

The findings of our systematic review and meta-analysis indicate that Ringer’s lactate solution is more effective than saline, HES, and albumin solutions in reducing all-cause mortality and improving hemodynamic parameters in critically hypotensive septic patients. Even though saline outperformed albumin and HES, due to the limitations of the included studies, further rigorous quality research is required for extensive comparison of different resuscitation fluid therapies and validation of these findings. Moreover, research into sepsis pathophysiology and innovative delivery methods would also enhance fluid resuscitation strategies, improving patient outcomes and quality of life.
